# Radiation hazards in PF-1000 plasma generator fusion research (part 3)

**DOI:** 10.1007/s10967-016-4700-1

**Published:** 2016-01-18

**Authors:** Kamil Szewczak, Slawomir Jednorog

**Affiliations:** 1 Central Laboratory for Radiological Protection, Konwaliowa 7, 03-194 Warsaw, Poland; 2 Institute of Plasma Physics and Laser Microfusion, Hery 23, 01-497 Warsaw, Poland

**Keywords:** Nuclear fusion, Health physics, Radiation protection, Radiometry, Neutrons

## Abstract

Plasma experiments conducted on the PF-1000 device generate the release of neutrons and ionizing radiation that are the source of immediate exposure to personnel. Neutron activation of materials in the research device and the surroundings is a source of ongoing radiation exposure to the same personnel. Having reported on personnel exposure from ionizing radiation and neutron activation, we now aim to characterize exposure from direct neutron emission generated by the device, and describe the process of ensuring measurement accuracy.

## Introduction

In nuclear fusion experiments mainly gaseous deuterium but occasionally deuterium and tritium are completely ionized into the plasma state. The kinetic energy of the nuclei overcomes the Coulomb repulsion, allowing nuclear fusion of hydrogen nuclei to take place.

Nuclear fusion research is commencing in three main directions. Magnetic confinement facilities (MCF) such as the tokamaks and stellarators are experimental models for future nuclear power plants. The Joint European Torus (JET) is the world’s largest MCF. It generates low-density plasma in large volumes. It is operated in Culham, Oxfordshire, UK. The toroidal vacuum vessel of the tokamak is surrounded by magnetic field coils whose strong field confines electrons and ionized nuclei. The first experimental tritium campaign on JET in 1991 resulted in a fusion power output of approximately 17 MeV. The International Thermonuclear Experimental Reactor (ITER) tokamak is located in Cadarache, Saint-Paul-lès-Durance, France. It is expected that the experience gained on this facility will be used in planning the Demonstration Power Plant (DEMO), a planned prototype power plant based on light element fusion. Both ITER and DEMO will harness the tokamak concept to plasma production.

The largest stellarator, the Wendelstein W7-X, is located in Greisvald, Germany. The W7-X is characterized by non-planar superconducting field coils, and, when in the steady state, can continue operation in the absence of strong electric current drive.

Inertial confinement facilities (ICF) are based on plasma generation by intense laser pulses. The largest ICF is the National Ignition Facility (NIF) at the Lawrence Livermore National Laboratory in Livermore, California, USA. NIF uses lasers to heat and compress a small amount of fuel consisting of isotopes of hydrogen, allowing nuclear fusion to occur.

Plasma-focus (PF) devices employ a high-energy electrical current to create plasma with isotopes of hydrogen, usually deuterium, within a sealed vacuum vessel. The current that occurs between the electrodes produces a magnetic field. Attractive Lorentz forces are generated across the electrodes, which narrow the deuterium stream in a so-called “pinch,” raising the temperature in the stream to allow fusion of the deuterons. The largest PF facility is installed in the Institute of Plasma Physics and Laser Microfusion (IFPILM) in Warsaw, Poland. The PF-1000 device is not designed to produce energy from nuclear fusion. It is a strong source of 2.5 MeV neutrons, up to 10^11^ per pulse, as well as of high-energy ionizing radiation, and is used for fundamental studies of dense magnetized plasma phenomena, and to test newly-designed neutron diagnostics systems dedicated to research on plasma and similar topics. The neutron yield changes with the successive pulses that the PF device is designed to produce. The majority of neutrons (up to 90–95 %) are generated during beam target phenomenon. The rest of the neutron yield is from thermonuclear synthesis reactions [1]. These emitted neutrons are potentially the most important source of radiation hazard to personnel.

We have already reported on the radiation safety challenges posed by the release of electromagnetic ionizing radiation, and by neutron activation of surrounding materials in the facility [2–6]. In this study we aimed to characterize the radiation hazard posed to personnel by neutron streams by the PF-1000.

The large facilities like JET, W7-X, NIF, and ITER have rigorous radiation protection programs, as well as systems of radiation shields designed along with the facilities. The PF-1000 radiation protection system has developed in step with our knowledge of its unique characteristics.

The PF-1000 device is located in a large laboratory space in the IFPILM. Movable paraffin wax panels covered with steel are used to protect personnel in the areas most frequently occupied, such as the steering room, where all the device controls are located. On the opposite side of the laboratory space is a Faraday cage with identical shielding. During experimental runs all researchers and technicians are in either the steering room or, occasionally, in the Faraday cage. Two the most exposed technicians that are permanently involved in PF-1000 research are provided with individual thermoluminescence dosimeters to monitor their exposure.

## Materials and methods

### Characterization of neutron exposure at the PF-1000 facility

The deuterium–deuterium (D–D) nuclear fusion reactions are presented in Eq. (1–3). Neutrons are the most hazardous radiation source in the laboratory space of the PF-1000 facility. They are generated in the first reaction (1):

1 $$d \, + \, d \, \to {\text{ 3He}} \left( {0. 8 1 7 {\text{ MeV}}} \right) \, + \, n \left( { 2. 4 5 2 {\text{ MeV}}} \right)$$

2 $$d\,+\,d \to t \left( {1.008{\text{ MeV}}} \right) \, + \, p \left( {3.025{\text{ MeV}}} \right)$$

3 $$d \, + \, d \to \alpha \left( {0.08{\text{ MeV}}} \right) \, + \, \gamma \left( {23.77{\text{ MeV}}} \right)$$

The products of the reaction presented in Eq. (2) are trapped inside the vacuum chamber walls. The generated 2.45 MeV neutrons (Eq. 1) leave the vacuum chamber and penetrate the surrounding environment, and are a source of occupational exposure to researchers involved in the experiments. A time-of-flight detector registers D–D neutrons up to 70 m from the PF-1000.

Various experiments on the PF-1000 may last from a few days to six months. During plasma research the vacuum chamber is filled with deuterium and up to 20 plasma discharges are fired per day [7].

### Neutron probe calibration

We evaluated a neutron probe LB6411^®^ (Berthold Technologies, Bad Wildbad, Germany) [8]. The active part of the dosimeter probe is a cylindrical proportional counter (40 × 100 mm) filled with a mixture of ^3^He and methane. This design, which mainly measures thermal neutrons, has a registration efficiency of approximately 90 %. The 25 cm diameter moderator that covers the probe, made from low-pressure polyethylene with a density of 0.95 g cm^−3^ doped with 2 % carbon increases its sensitivity for non-thermal neutrons with different energies. This allows registration of neutrons with energies up to 20 MeV. According to the instrument specifications, the number of registered pulses is proportional to the ambient dose equivalent, H*(10)_*n*_. The manufacturer guarantees that the probe measures H*(10)_*n*_ over a range of 100 nSv h^−1^–100 mSv h^−1^ with a range of uncertainty of 30 %. The response function of the instrument is nonlinear and strongly depends on neutron energy [8]. The probe is supplied with a fixed internal conversion factor evaluated for neutrons emitted by ^252^Cf (*W*_p_^Cf^ = 353 pSv pulse^−1^).

To validate the internal conversion factor supplied by the manufacturer, we calibrated the probe using neutrons from an ^241^Am-Be source at a facility certified by the Polish Center for Accreditation. Two reference values of ambient dose equivalent were checked during the test. According to the probe specifications, the conversion factor for neutrons from an ^241^Am-Be source is *W*_p_^AmBe^ = 337 pSv pulse^−1^. We sought to validate the ratio of the abovementioned factors [(W_p_^AmBe^)(W_p_^Cf^)^−1^].

Next, because we work in a mixed *n* + γ environment, we assessed the probe’s sensitivity to electromagnetic radiation using a γ radiation field emitted by ^137^Cs with an ambient dose equivalent rate of 16,827 μSv h^−1^.

### Neutron energy spectra

**Fig. 1:**
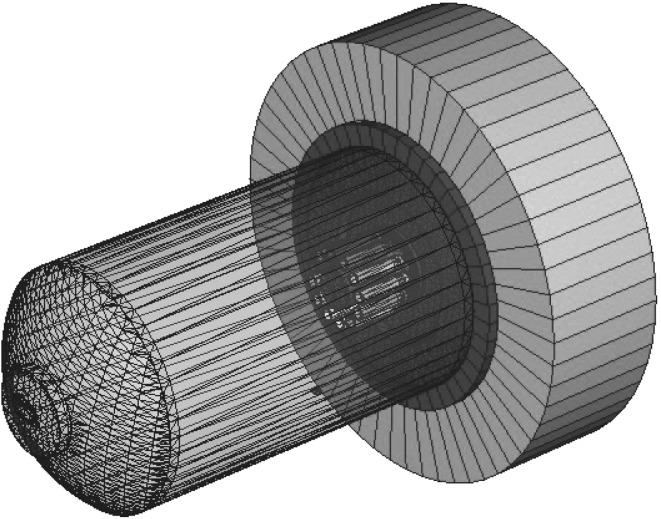
The geometrical model of the PF-1000 plasma generator. It allows Monte Carlo N-Particle (MCNP) calculation of neutron energy distribution in the reference positions

Our next goal was to assess the conversion factor’s adequacy for quantifying neutrons with the energy spectra characteristic of the D–D reaction and different positions occupied by researchers and technicians during research runs. To assess for appropriate conversion factors for the position-dependent neutron energy spectra the PF-1000 generates, we simulated the spectra using the Monte Carlo N-Particle (MCNP) numerical technique, applying the MCNP5 code [9]. The geometry outlines in the input file include the PF-1000 as the main scattering/absorbing element of the facility, most importantly, the vacuum chamber of the PF-1000 (Fig. 1). The concrete walls and floors and the neutron shields, as well as the walls of the control room and Faraday cage in the laboratory space, were also included in the geometrical input.

In this test we assumed that the neutrons are emitted from a point source but their energy depends on the direction of emission. This takes into account the beam-target phenomena inherent in the D–D plasma generation with PF devices [7]. Each simulation procedure observed 2 × 10^8^ interaction events caused by single neutrons. We derived the neutron fluence using four spherical detectors each witch 10 cm in diameter using the point detector tally (F5). The result was normalized to the total neutron fluence at the designated points.

### Ambient neutron dose equivalent assessment

Although the PF-1000 is equipped with some neutron diagnostics, including a silver activation counter, a fast neutron yttrium monitor [10], and a beryllium counter [11], they are not suitable for neutron dosimetry purposes.

Using the recommendations of the International Commission on Radiological Protection (ICRP) [12], we set the ambient dose equivalent H * (10)_n_ as an approximation of the effective dose.

Neutron doses were monitored in four reference positions: two inside the laboratory space (the steering room, position 1), (the Faraday cage, position 2), and two in adjoining rooms (the assembly hall, position 3), (the visitors’ room, position 4). The steering room is located behind the PF-1000, four meters from its long axis and four meters from the device’s collector. The visitors’ room is approximately ten meters from the back of the device and two meters from the main device axis but in a different part of the building, separated from the laboratory space by a concrete wall. The Faraday cage is the location closest to the PF-1000, three meters from the front port and two meters from its long axis. The assembly hall is located anterior to the device, but in another part of the building, separated by a light brick wall.

The measurements were conducted in single pulse measurement mode, giving uncorrected values of H*(10)_*n*_. These values then had to be corrected according to the neutron energy spectrum in the position being interrogated. We used corrected conversion factors according to recent methodology [13].

The final value of the ambient dose equivalent was calculated according to the formula

(4) $$H ^{\star} \left( {10} \right)_{n}^{i,j}\, =\, \frac{{H ^{\star} \left( {10} \right)_{n}^{{i,j,W_{p}^{\text{Cf}} }} }}{{W_{p}^{\text{Cf}} }} \times W_{p}^{j} ,$$

where H*(10)_*n*_^*i*,*j*^ is the ambient dose equivalent corrected value for the ith discharge measured in the jth position, $H ^{\star} \left( {10} \right)_{n}^{{i,j,W_{p}^{\text{Cf}} }}$is the value indicated by the instrument, *W*_*p*_^Cf^ is the internal calibration coefficient of the instrument, and *W*_*p*_^*j*^ is a corrected conversion factor for the jth position.

Using the coefficients proposed by ICRP [14], we recalculated the doses expressed as ambient dose equivalents to effective dose. The values of the coefficients were 0.20 for positions 1 and 2 and 0.65 for position 3. No neutrons were detected at position 4.

The corrected measurement values were coupled with the neutron yield measured by the installed silver activation monitors [11]. Basing on the dependence of H*(10)_*n*_ on a particular discharge and the annual neutron yield [2, 3], the annual ambient dose equivalent was estimated for the particular positions over the period from 2001 to 2013. The final values of effective doses correspond to the situation where personnel are situated in the specific positions during all realized discharges [4, 6].

## Results and discussion

### Results of the LB 6411 probe calibration

The results of our measurements using the neutrons from the ^241^Am-Be source show that the calibration factor is constant (Table 1).

**Table 1:** Results of testing the LB6411 probe in an ^241^Am–Be neutron beam

Reference values, H*(10)_*n*_^*r*^ [µSv]	Measured values, H*(10)_*n*_^*m*^ [µSv]	Calibration factor, *k*
15.50	15.95	0.97
8.02	8.27	0.97

The result obtained at the first step of radiometer calibration proves the validity of the internal conversion factor provided by the manufacturer. At the second step of the calibration procedure, i.e., during exposure of the instrument to γ-radiation, when the instrument was exposed in a field at an ambient dose equivalent rate of 16,827 µSv h^−1^, the probe indicated 1.03 µSv h^−1^. Thus the discrimination factor is approximately 6.1 × 10^−5^, indicating its suitability to be operated in a mixed *n* + γ radiation field.

**Fig. 2:**
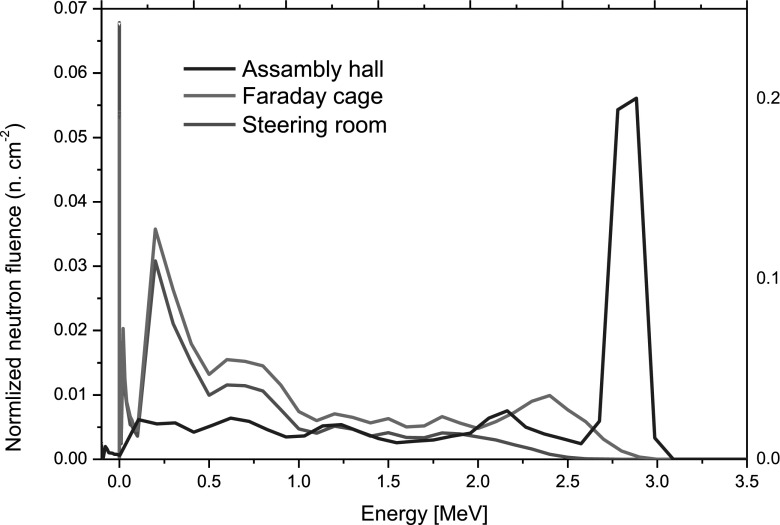
Normalized neutron fluence spectra evaluated for all measured positions subject to exposure. Normalized neutron fluence is the quotient of neutron fluence that is generated by a single neutron source during a neutron emission divided by the total neutron emission (taken as the reading on the neutron monitor that is calibrated to measure the total number of neutrons generated during plasma discharges). Both quantities are expressed in the same units

Figure 2 displays the results of MCNP simulation for the three locations with measurable neutron exposure. Because radiation shields separate these locations from the neutron emission source, the main contribution to the total neutron fluence at positions 1 and 2 are from thermalized neutrons. In position 3, neutrons with energies of approximately 2.8 MeV are the most likely source. The 2.45 MeV neutrons that are generated during D–D fusion (see Eq. 1) are indicated only in the system’s center of mass. The 2.8 MeV neutrons are detected in a laboratory system that takes into account also the motion of the deuterium ions that were the projectiles during collisions with deuterium target. For energy conservation it is also necessary to take into account the target velocity. The assembly hall lies in the neutrons’ path with no intervening shielding.

The values of the corrected conversion factors for each position are presented in Table 2. In the case where the main contribution to the measurement is from thermal neutrons, the conversion factor increases more than twice compared with the conversion factor fixed in the instrument.

**Table 2:** The corrected conversion factors measurements at each position

Position no.	Corrected conversion factors *W*_*p*_^*j*^ [pSv pulse^−1^]
control room (*j* = 1)	693
Faraday cage (*j* = 2)	606
assembly hall (*j* = 3)	351

The relations shown by Eqs. 5–7 describe the linear dependence of the corrected ambient dose equivalent values on neutron yield. The last digits in each equations indicate the lowest values of the neutron yield for which the method is applicable. Consequently for the position 1, 2 and 3 these values are *Y*_*n*_ = 1.97^9^, 8.17^10^ and 5.78^9^.

*Position 1:*

5 $$H\, ^{\star}\,(10)_{n}^{i,1} = 7.8 \times 10^{ - 10} \times Y_{n}^{i} - 1.5,$$

*Position 2:*

6 $$H\,^{\star}\, (10)_{n}^{i,2} = 2.9 \times 10^{ - 10} \times Y_{n}^{i} - 23.7,$$

*Position 3:*

7 $$H\,^{\star}\, (10)_{n}^{i,3} = 8.3 \times 10^{ - 10} \times Y_{n}^{i} - 4.8.$$

#### Annual effective neutron doses

**Fig. 3:**
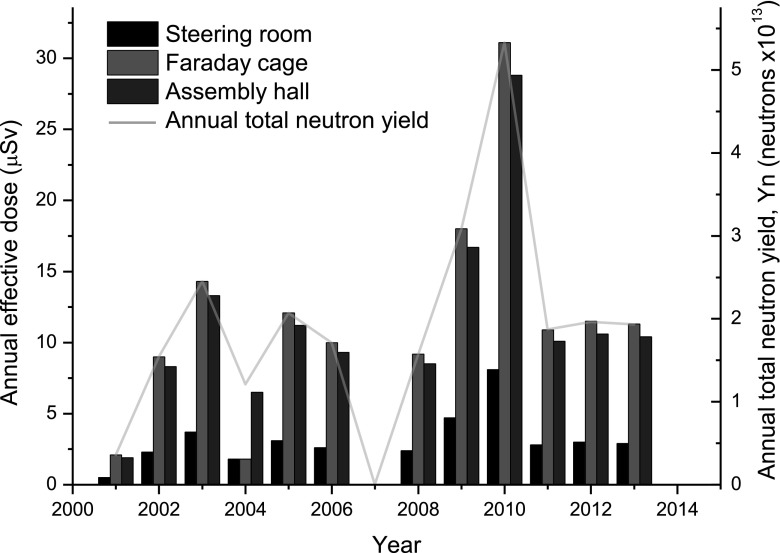
Annual effective doses from neutrons from D–D fusion to personnel located in three different parts of the laboratory space subject to exposure during operation of the PF-1000

Figure 3 shows the annual effective doses for the positions occupied by personnel over the years 2001–2013. This same graph displays the neutron budget over subsequent years. One can easily conclude that doses are linearly dependent on the neutron budget. This is in accordance with Eqs. 5–7. The absence of results for 2007 is due to PF-1000 maintenance downtime.

### Accuracy of neutron radiation hazard assessment

The accuracy of our neutron dose metrology depends on the Berthold radiometer calibration and evaluation of corrected conversion factors that indirectly depend on MCNP calculation accuracy and accuracy of measuring the distance from the source. The highest uncertainty range, 30 %, is attributable to the Berthold radiometer. The corrected conversion factor evaluation was performed with an accuracy of 0.1 % (see Table 1). The MCNP calculation accuracy depends mainly on the accuracy of the geometrical input preparation and numbers of evaluated histories. From our experience, the simplifications that had to be implemented in case of such a complex device did not influence neutron spectra predictions for observed positions. The accuracy of the calculation process was kept to the level of 1 %. When assessing personnel exposure at the different positions, we used a “worst-case scenario” hypothesis regarding length of exposure, distance from the source, and working time. That could lead to an overestimation of the radiation dose, but not the neutron hazard, as it was evaluated based on the neutron budget.

## Conclusions

- It is possible to use a commercial probe for neutron occupational exposure assessment during nuclear fusion research and other plasma experiments using the PF-1000 but The probe must be intensively assessed before its application.
- The methodology used included instrument calibration and neutron spectra simulation using Monte Carlo methodology.
- Combining the information of the probe’s sensitivity for neutrons with different energies and the simulation results, we were able to arrive at corrected conversion factors adequate for measurements at the different locations.
- We proved that the correct selection of the above mentioned factors has a significant influence on the final results. The estimated corrected conversion factors were more than 2 × the internal conversion factor specified by the manufacturer and are valuable for neutrons of different energies from the D–D neutrons.
- The neutron shields mounted around the PF-1000 have been properly designed and they significantly decrease the energy of passing neutrons.
- The maximum annual effective dose to the Faraday cage in 2010 was 31.1 µSv. That value shows that the neutrons make no significant contribution to the total annual effective dose for personnel operating the PF-1000.
- The neutron doses were significantly below annual limits, even factoring in the 30 % inaccuracy of the probe [8].
- Radiation dosimetry needs to be carefully assessed for every plasma experiment, especially with varying experimental conditions.
